# *In situ *methods to localize transgenes and transcripts in interphase nuclei: a tool for transgenic plant research

**DOI:** 10.1186/1746-4811-2-18

**Published:** 2006-11-02

**Authors:** Ana Paula Santos, Eva Wegel, George C Allen, William F Thompson, Eva Stoger, Peter Shaw, Rita Abranches

**Affiliations:** 1 Plant Genetic Engineering Laboratory, Instituto de Tecnologia Química e Biológica, UNL, Av. República, 2781-901 Oeiras, Portugal; 2 Department of Cell and Developmental Biology, John Innes Centre, Colney, Norwich NR4 7UH, UK; 3 Plant Transformation Laboratory (PTL), Departments of Crop Science and Horticultural Science, Campus Box 7550, North Carolina State University, Raleigh, NC 27695, USA; 4 Plant Gene Expression Laboratory, Campus Box 7550, North Carolina State University Raleigh, NC 27695, USA; 5 Institute for Molecular Biotechnology, RWTH Aachen, 52074 Aachen, Germany; 6 Plant Cell Biology Laboratory, Instituto de Tecnologia Química e Biológica, UNL, Av. República, 2781-901 Oeiras, Portugal

## Abstract

Genetic engineering of commercially important crops has become routine in many laboratories. However, the inability to predict where a transgene will integrate and to efficiently select plants with stable levels of transgenic expression remains a limitation of this technology. Fluorescence *in situ *hybridization (FISH) is a powerful technique that can be used to visualize transgene integration sites and provide a better understanding of transgene behavior. Studies using FISH to characterize transgene integration have focused primarily on metaphase chromosomes, because the number and position of integration sites on the chromosomes are more easily determined at this stage. However gene (and transgene) expression occurs mainly during interphase. In order to accurately predict the activity of a transgene, it is critical to understand its location and dynamics in the three-dimensional interphase nucleus. We and others have developed *in situ *methods to visualize transgenes (including single copy genes) and their transcripts during interphase from different tissues and plant species. These techniques reduce the time necessary for characterization of transgene integration by eliminating the need for time-consuming segregation analysis, and extend characterization to the interphase nucleus, thus increasing the likelihood of accurate prediction of transgene activity. Furthermore, this approach is useful for studying nuclear organization and the dynamics of genes and chromatin.

## Background

The production of transgenic plants is now routine for many crop species and different technologies for gene transfer are available for a wide number of species, including some previously thought to be recalcitrant to transformation. However, the unpredictability of integration sites and lack of expression stability are still limitations in plant transgenic technology. Significant efforts have been made to understand the mechanisms of transgene integration in the host genome (reviewed in [1]). Most studies have focused on characterizing transgene loci by sequencing or genetic dissection of the integration sites. Cell biology techniques have been used to complement these molecular approaches and a great deal of information has been obtained from the visualization of transgenes by fluorescence *in situ *hybridization (FISH) on metaphase spreads (e.g. [2-11]). This technique makes it possible to physically map transgene integration sites, but complex patterns of integration are often found in transgenic plants and the underlying mechanisms of transgene integration are still far from being completely understood. Since predictable transgene expression is the goal, it is important to remember that most gene transcription takes place during interphase and not metaphase. We believe that visualizing transgenes and their transcripts in interphase nuclei can provide information about transcriptional competency, and that this knowledge can be used to improve prediction of transgene behavior.

There is now good evidence that the spatial organization within the cell nucleus has a strong impact on gene expression (reviewed in [12,13]). Our previous work has shown that FISH on three dimensional nuclei of transgenic plants can provide new insights into the relationship between higher order chromatin structure and the expression of endogenous genes and transgenes [14-16]. We have shown that FISH can be used to better understand chromatin and gene organization and dynamics by following the localization of transgenes during the cell cycle [2], or by inducing architectural modifications of chromatin [14]. A number of published studies show that spatial clustering of endogenous sequences affects gene expression (reviewed in [13,17]; [18,19]). Sproul et al. [20] reviewed how chromatin structure can control not only the expression of individual genes, but also the simultaneous regulation of multiple genes, in organisms such as yeast, Drosophila and *C. elegans*. The ribosomal genes are the best characterized example of gene clustering in plants (e.g. [21]). However, the concept of gene clustering can be extended to include transgene repeats that are integrated as multiple copies, such as we have observed in wheat [2]. FISH has proved to be an important tool for understanding the behavior of genes (endogenous or exogenous) and how they are regulated within the context of nuclear organization (reviewed in [22]). FISH can also be a valuable tool for dissecting the complex mechanisms of transgene integration in the host genome. The importance of understanding the factors that influence higher order transgene organization in order to optimize transgene expression has been reviewed in [23]. Knowledge of these factors will improve manipulation of transgene expression stability, and thus has implications both in fundamental and applied research.

In this paper we review a set of techniques that allow the use of FISH to visualize transgene integration sites in interphase cells. We describe the preparation of whole tissue sections where the 3D structure of the nucleus is well preserved, isolated nuclei in which probe penetration is facilitated, and histone-depleted nuclear halos in which the arrangement of transgene insertions can be visualized in more detail relative to the nuclear matrix or DNA loop domains. We also describe the use of extended DNA fibers, in which it is possible to observe short genomic regions interspersed with repeated transgenes, and the localization of transgenic RNA by FISH using RNA probes. Since previous applications of FISH have focused on highly condensed, transcriptionally inactive metaphase chromosomes, we compare the information obtained from metaphase FISH to that using interphase nuclei. It is clear that both applications of FISH provide important information that complements the data from traditional techniques such as Southern hybridization and PCR. The techniques that have been developed allow the visualization of single copy transgenes and their transcripts in several different plant species. The implications of our results are discussed both from the applied perspective, for determining the likely stability of transgene expression, as well as how they may increase our fundamental knowledge of the relation between nuclear structure and gene expression.

## Spatial organization of the transgene locus is important for predicting transgene expression and stability

The genomes of several model and crop species have now been fully sequenced, or will be in the near future. This information opens up new opportunities for dissecting pathways that determine the successful expression of integrated transgenes and will bring us closer to being able to manipulate transgene expression and stability. Over the last few years, our view of how genes are regulated has expanded from a focus on DNA sequences (e.g. promoters and enhancers) to a broader appreciation of chromatin-mediated regulation of gene expression. Accumulating knowledge on the histone code [24,25], or even more recently, the "chromatin code" [26-28], has provided information on multi-protein complexes that directly or indirectly affect the chromatin structure around a gene. Epigenetic information has a major role in the control of gene expression and has been related to physical changes in the organization of the locus. Major epigenetic modifications of chromatin include cytosine methylation [29,30] and several key post-translational modifications of histones [28]. The best studied histone modification is the acetylation of histone tails [31,32]; a more 'open' chromatin state resulting from histone acetylation is thought to increase the accessibility of transcription complexes to genomic DNA [33]. The control of chromatin structure is complex and also involves mechanisms such as RNA interference (originally termed post-transcriptional gene silencing) and transcriptional silencing. However a detailed discussion of this topic is beyond the scope of this review.

In transgenic plants, the effect of epigenetic modifications on transgene expression is most evident when independent transformants carrying the same transgene show different levels of expression even when inserted at the same genetic locus [34]. There is now evidence that many of these differences are a consequence of biochemical differences in chromatin at the different integration positions [26]. To date, most reports on transgene locus structure and organization have been obtained using molecular tools, such as PCR, sequencing and southern blot analyses, which provide information about the structure and location of the transgene integration site, although some reports have included studies on metaphase spreads, with chromosomal mapping of transgene loci using FISH. Identification of the chromosome and chromosomal regions where the transgene has been integrated provides important information on the site of transgene integration, as well as possible differences in locus structure between different methods of transformation. This type of analysis has shown that there is no preference for integration in particular chromosomes when particle bombardment is used in wheat [2], barley [7] or oat [35] transformation, but there is often a preference for integration in the distal regions of the chromosome arms. For example, the majority of transgene loci in petunia [36,37] and oat ([8], [35] and references therein) are localized in telomeric or sub-telomeric regions. Iglesias et al. [38] used FISH to probe the physical location of transgene insertion in tobacco, and demonstrated that the stably-expressed inserts were in the vicinity of telomeres, whereas the unstably-expressed inserts occupied intercalary and paracentromeric locations.

We have collective experience with several plant species: wheat, rice, tobacco and Arabidopsis, as well as tobacco suspension cell cultures. Other investigators have studied many other species. This has provided a wide variety of material to study several aspects of chromosome and (trans)gene organization and expression. We have previously observed clustering of transgenes in the interphase nucleus of plants containing multiple transgene integration sites, which was not evident in metaphase chromosomes. We hypothesized that the transgene sites were brought together in interphase nucleus because they were recruited to a common functional domain such as a transcription factory, perhaps reflecting the fact that they shared the same promoter [2]. This demonstrates the importance of using FISH on interphase nuclei to obtain information that could not be obtained by any other method. More recently, and mostly in literature on animal systems, it has been shown that chromatin structure has a role in regulating the expression of clustered genes (reviewed in e.g. [20]). Multiple tandemly integrated copies of transgenes are often generated by particle bombardment and may affect the structure of the chromatin surrounding the transgenes, which may in turn affect their expression and/or stability. Interestingly, introduced DNA lacking genes can also be condensed into heterochromatin. Pecinka et al. [39] showed that arrays of repeated *lac *operators used for Green Fluorescent Protein tagging of DNA had higher frequencies of association with each other and with heterochromatin than expected, which may alter the spatial chromatin organization in the nuclei. In the recent years it has become clear that molecular analysis, along with phenotypic and genotypic segregation analyses, are not sufficient to fully understand the complexity of transgene loci. We therefore suggest that it is essential to look at interphase nuclei to get a deeper understanding of the role of nuclear structure in the regulation of transgene expression.

## Fluorescence *in situ *hybridization (FISH) is essential for a full characterization of transgenic plants

Transgene loci vary in size and complexity, and the site of integration may have properties that favor integration or selectable marker expression. *Agrobacterium*-mediated transformation results in a higher proportion of simple inserts than is produced using microprojectile bombardment. FISH of transgene loci on metaphase and pro-metaphase chromosomes [2,11,35] and on extended DNA fibers ([40], Wegel and Shaw, unpublished]) shows that genomic interspersions in between multiple transgenes can vary in length from a few kilobases to several megabases. The presence of complex transgene loci suggests that these loci may also exhibit some level of transgene scrambling because transgene locus formation appears to proceed via Illegitimate Recombination (IR) [8,23,41] regardless of the DNA delivery method. Transgene scrambling can cause problems with gene expression because complex transgene loci are often associated with transgene silencing. Understanding the processes that occur during integration is more likely to lead to strategies for producing stably expressing transgenic plants. As the sensitivity of FISH techniques improves, more information can be gained and integration events can be better characterized.

### Sensitivity of FISH for in situ detection of transgenes – detection of single-copy genes

Since the first description of *in situ *hybridization in 1969 [42], many advances have been made in the sensitivity of detection of DNA and RNA molecules at the cellular and subcellular levels. FISH has come to be used frequently as a tool in basic and applied research because detection is sensitive and allows discrimination of multiple targets in the same sample. The efficiency and sensitivity of FISH depends on the accessibility of the cytological targets and the size of the probes. In FISH, as with most labeling techniques in cell biology, there is a compromise between optimal preservation of cell and tissue morphology and accessibility to labeling reagents [43]. Thus the need for good ultrastructural preservation may limit the sensitivity of FISH for mapping DNA sequences on plant chromosomes. In general the target DNA sequences have been limited to large or high copy number inserts from 10 to 60 kb (e.g. [35,44]). Technical difficulties in detecting single or low copy number target sequences are partly due to the large proportion of non-target repeat sequences, which in some cases are more than 90% of the genome [45]. There are some reports on the detection of small, single copy DNA sequences in plants; table 1 includes examples where single or low copy number transgenes and some endogenous genes have been analyzed by FISH. For example, a successful detection of one or two copies of T-DNA to metaphase chromosomes of *Petunia hybrida *was performed using a 2.7 Kb probe [46]. Detection of targets as small as 4 Kb in maize interphase nuclei has also been reported [47]. To date, the shortest reported unique DNA sequence localized on mitotic plant chromosomes is 684 bp in *Beta vulgaris *[48].

**Table 1:** Sensitivity of FISH to detect single or low copy genes or transgenes.

**Plant Species**	**Target DNA**	**Phase of Cell Cycle**	**Probe size**	**Reference**
*Petroselium crispum*	Endogenous	Metaphase	6,6 Kb	[104]
*Oryza sativa*	T-DNA	Metaphase	5,6 Kb	[3,105]
*Oryza sativa*	T-DNA	Metaphase	5,5 Kb	[106]
*Zea mays*	T-DNA	Meiosis (pachytene)	3,1 Kb	[107]
*Zea mays*	Endogenous	Meiosis (pachytene)	3,1 Kb	[108]
*Petunia hybrida*	T-DNA	Metaphase	2.7 Kb	[46]
*Triticum aestivum*	Bombardment	Interphase nuclei and metaphase chromosomes	1.8 Kb	[2,14]
*Asparagus officinalis*	Endogenous	Interphase nuclei and metaphase chromosomes	1,4 Kb/1,7 Kb	[109]
*Oryza sativa*	Endogenous	Metaphase	1,29 Kb	[110]
*Petunia hybrida*	T-DNA	Metaphase	4 Kb	[36]
*Allium cepa*	T-DNA	Metaphase	710 bp	[49]
*Beta vulgaris*	Endogenous	Metaphase	684 bp	[48]

Methods such as Tyramide-FISH, in which signals can be amplified by the enzymatic deposition of fluorochrome-conjugated tyramide, have been adapted for plants and target sequences as small as 710 bp on *Allium cepa *mitotic chromosomes have be detected [49]. Another approach to increase FISH sensitivity is the use of primed *in situ *DNA labeling (PRINS, [50]). PRINS uses a primer-based amplification of the target DNA in a chromosomal preparation containing fluorescent-labeled nucleotides. Menke et al. [51] have compared the sequence resolution of PRINS versus FISH on plant chromosomes and found that PRINS was useful for the detection of high copy number repeats, but could not be used to detect a low copy number gene family. A more sensitive modification, called cycling-PRINS (C-PRINS), has since been developed. This technique includes the use of thermal cycling, similar to PCR, and has been reported to be able to detect low copy number repeats [52,53].

Increasing the sensitivity of photometric detection has also allowed the visualization of smaller sequences. For example, the use of a cooled charge-coupled device (CCD) camera can increase the detection sensitivity 30-fold compared with simpler digital cameras or film. For DNA-FISH, unique sequences of 1–2 kb can be detected on metaphase chromosomes with a resolution of about 3 Mbp. With FISH on highly decondensed chromatin (i.e. naked DNA fibers) a sensitivity of 200 bp and a genomic resolution of about 1 Kb can be obtained. However the efficiency of DNA-FISH decreases as the target DNA becomes smaller. The intensity of signals from small targets indicates that the sensitivity of DNA-FISH is only in part determined by the ability to generate sufficient photons for detection. Other factors such as accessibility, DNA loss, and *in situ *renaturation of the DNA target and probe sequences are equally important. In chromosomal and fiber-FISH a considerable level of noise is manageable as the specificity of the signals can often be verified by positional information.

## FISH can be applied to distinct cytological targets

The sensitivity and resolution of FISH on interphase chromatin depends on the cytological target it is applied to, and mainly on the state of chromatin condensation (see reviews [54-56]). We have performed FISH on interphase nuclei with progressively lower levels of chromatin compaction: (1) well preserved 3D structures in thick root tissue sections prepared with a vibratome; (2) isolated nuclei that maintain their 3D structure although extracted from a tissue; (3) histone depleted nuclei or nuclear halos; and (4) extended DNA fibers.

### FISH in interphase nuclei of 3D well preserved tissue sections

Vibratome tissue sections analyzed by confocal microscopy have been used to study the 3D organization of centromeres, telomeres, chromosomes, transgenes and other genomic sequences contained within bacterial artificial chromosome probes (BACs) in interphase nuclei of wheat tissues e.g. anthers, roots, endosperm and embryos [2,14,16,43,57,58], rice root tissue [59,60], tobacco root tissue [Abranches, unpublished] and Arabidopsis roots [61]. In all cases good preservation of the tissue structure has been achieved. For sectioned material, most fixatives are based on agents such as formaldehyde. A small amount of glutaraldehyde is sometimes added to the fixative; however the glutaraldehyde concentration should be kept low (0.05–0.1%) as it may induce autofluorescence in the tissues. Extended periods of fixation or high concentration of fixative may reduce accessibility, so a compromise must be made in order to preserve adequate structure while retaining accessibility [62]. Transgenic DNA can be detected by FISH using labeled DNA as a probe in tissue sections. The probe needs to penetrate into the tissue and gain access to the interior of the nuclei. Thus, several pretreatments must be performed and these will depend on the tissue type and on the species. The size of the probe is also crucial; the optimal size of DNA fragments for labeling is between 100–500 bp. For specific targeting of the transgene sequence, it is possible to use the isolated coding sequence of the transgene for labeling instead of the whole plasmid ([5]; Abranches, unpublished results).

**Figure 1:**
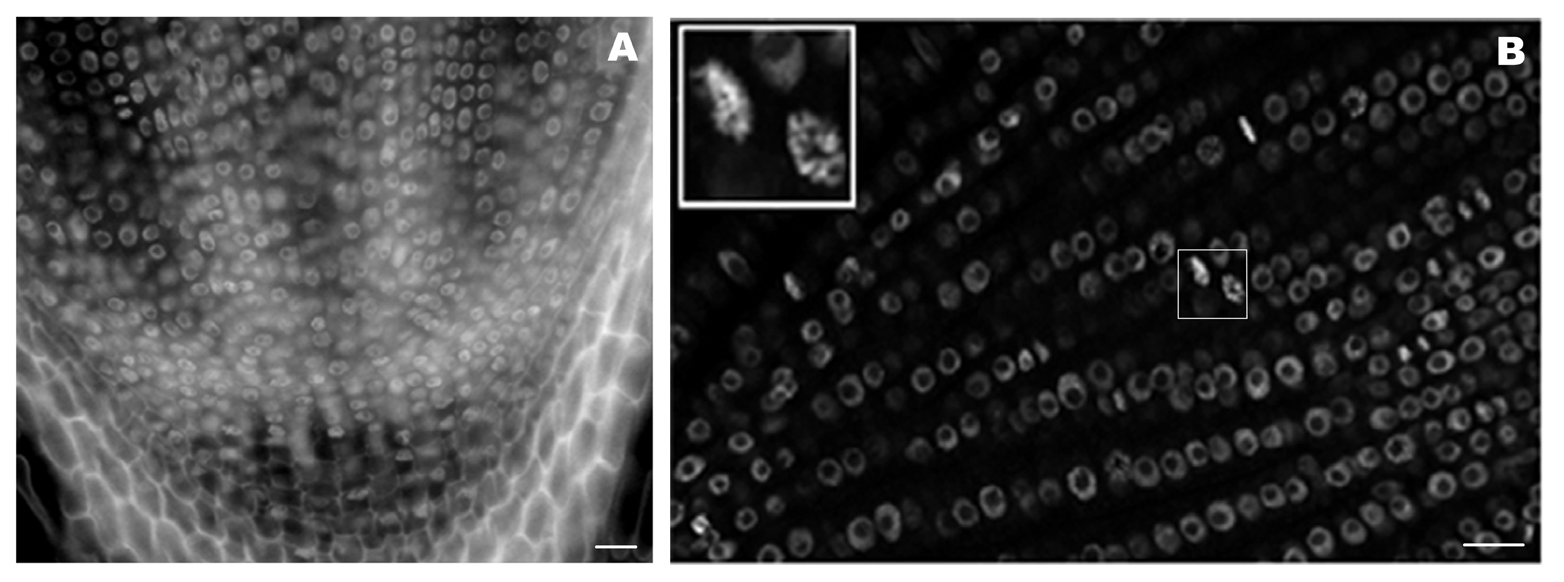
Rice root tissue section labeled with DAPI. The vibratome longitudinal section is 20 μm thick, containing about 2 cell layers. In (A) the entire section is shown while in (B) a single confocal section is shown. Note the inset in (B), which shows cell division stages. Bar, 20 μm.

**Figure 2:**
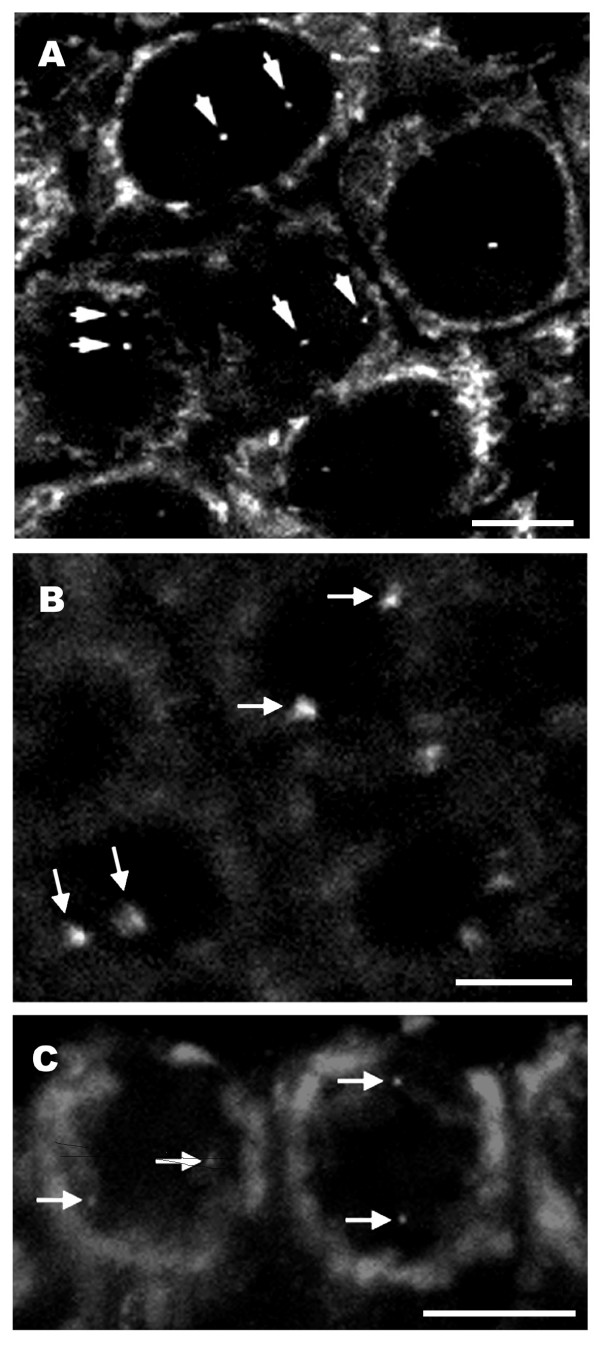
Transgene sites in 3D interphase nuclei of wheat (A), rice (B) and tobacco (C) root tissue sections. The wheat line (A) is homozygous and carries two transgene copies per homologue at a single site in the metaphase chromosome [2], each homologue is indicated by arrows. The rice line (B) was labeled with the plasmids SCM11, K1, H28, J1 [64], which were all co-bombarded. The tobacco line (C) contains 7 copies of the GUS gene and is a double haploid [63]. Confocal image stacks were recorded with a section spacing of 1 μm and a projection of two confocal sections is shown. Hybridization signals are indicated by arrows and show two single dots one for each homologue. Each dot can include multiple copies of the transgene. Bars, 10 μm.

In figure 1 we show a fixed 20 μm thick rice root section prepared with a vibratome. The DNA is stained with 4',6-diamidino-2-phenylindole (DAPI). The root-tip was previously fixed with 4% formaldehyde, freshly made from paraformaldehyde, and the tissue structure is well preserved. Figure 2 shows FISH images of transgenic DNA from root tissue sections from different plant species, including wheat (2A), rice (2B) and tobacco (2C). The wheat line in figure 2A contains only two copies of the GUS gene [2]. The two copies can be seen as a single signal within the nucleus. Thus each nucleus contains two dots, each one corresponding to one of the homologous chromosomes (Fig. 2A). This demonstrates the high sensitivity of the technique. The tobacco line shown in Figure 2C contains 7 copies of the GUS gene [63]. The transgenic rice line shown in figure 2B is more complex and produces four transgene-derived polypeptide chains [64], which result from co-transformation using four separate plasmids encoding four antibody components (the secretory component; the light chain; the heavy chain; and the joining chain), resulting in the assembly of a secretory antibody. FISH probes were prepared from a mix of the four plasmids that were used in co-transformation. When lines are homozygous, an even number of spots is visible. Interestingly, two discrete transgenic loci are seen in figure 2B. The arrangement of transgenes as well as their relative position is also informative, as has been discussed previously [2,14]. For studies on thick tissue sections it is preferable to use a confocal microscope, which allows for a detailed analysis through the depth of the tissue and subsequent 3D reconstruction of the nuclei.

### Isolated interphase nuclei of plants or plant cell lines

**Figure 3:**
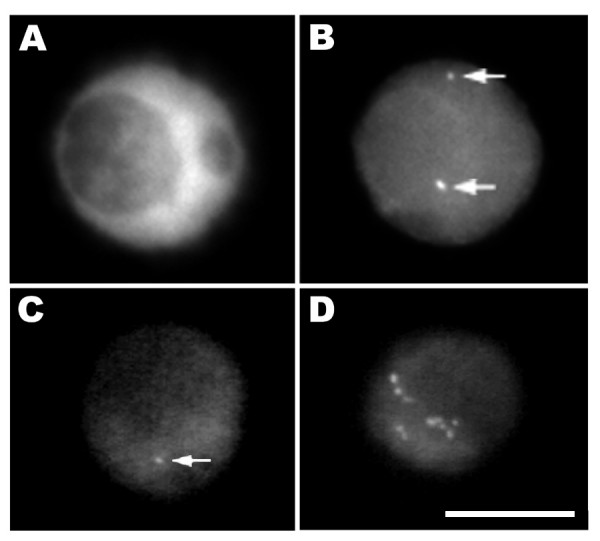
Transgene sites visualized in tobacco isolated nuclei. In panels A and B a single nucleus is shown, stained with DAPI (3A), and labeled by FISH (3B). This nucleus originates from a double haploid tobacco transgenic plant line that contains 7 copies of the GUS gene [63]; two signals, each corresponding to a homologous chromosome, are clearly visible (arrows). In panels C and D two isolated nuclei from independent NT1 tobacco suspension cell lines expressing luciferase are shown [65]. Panel C demonstrates the detection of a single copy luciferase gene using a *luc *fragment probe. Panel D shows a nucleus with multiple insertions in a total of 48 transgene copies. Bar, 10 μm.

Good preservation of nuclear structure is also obtained for isolated nuclei made by chopping the plant tissue with a razor blade in a suitable stabilizing buffer. In this method, the nuclei are spun onto a glass slide where they adhere and retain much of their 3-dimensional organization, as can be confirmed by confocal microscopy analysis. The same procedure can also easily be applied to cell suspension cultures, which divide rapidly compared to most plant tissues. This is a very informative approach for studying cell cycle changes. In figure 3, isolated nuclei from transgenic tobacco plants visualized with a CCD camera are shown. In panels 3A and 3B a single nucleus is shown, stained with DAPI (3A), and labeled by FISH (3B). This nucleus originates from a double haploid tobacco transgenic plant line that contains 7 copies of the GUS gene per haploid genome [63]. Two spots are visible, presumably corresponding to a single locus on each of a pair of homologous chromosomes. In panels 3C and 3D we show other isolated nuclei from NT1 tobacco suspension cell lines expressing luciferase [65]. Different transgene integration patterns are shown. Figure 3C demonstrates the detection of a single copy luciferase gene using a *luc *fragment probe in a cell line from [65]. Figure 3D allows us to make a comparison with a cell line containing 48 copies of luciferase, as estimated by competitive PCR [66].

### Histone-depleted nuclear halos

**Figure 4:**
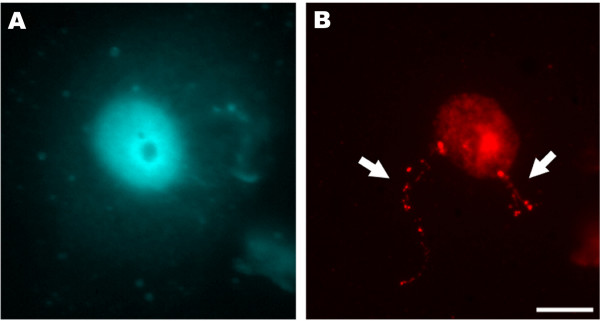
Transgene sites visualized in nuclear halos from a tobacco plant line, which contains 60 transgene copies [63]. A) DAPI staining, B) FISH signals clearly visible as two strings of dots. Nuclear halos were produced by treatment with LIS (lithium diiodo salicylate) which removes soluble proteins, including histones. The DNA can be seen as a halo surrounding a residual nucleus. In isolated nuclei, two signals, each corresponding to a homologous chromosome, are clearly visible (arrows). In contrast, in nuclear halos, a strand like arrow of signals is clearly visible. Bar, 10 μm.

DNA halo preparations are obtained from interphase nuclei (on microscope slides). In this method DNA loops are formed by selectively removing the histones, producing a nuclear halo around the residual nuclear matrix in which the bases of loops remain attached to the matrix [40,67-69]. The two most commonly used methods for removing the histones to prepare nuclear halos are either a high-salt extraction [70] or a detergent extraction using lithium 3'-5'-diiodosalicyclic acid (LIS) [71]. The arrangement of transgenic sites can be analyzed on these DNA loops providing important information on where the transgene has integrated in the context of the DNA loops that are anchored to or associated with the matrix. Some of the tobacco transgenic lines used in our studies contain a reporter gene flanked with the RB7 MAR [63,72] which might alter matrix association. We performed FISH on nuclear halos using the transforming plasmid as the probe and collected images using a CCD camera. Figure 4 shows a nuclear halo prepared from the root tip cells of a tobacco line containing 60 copies of the transgene. In figure 4A, the DNA is stained with DAPI and the faintly fluorescent dispersed DNA fibers are seen as a halo spreading outside the brightly stained residual nucleus. In figure 4B, the FISH signal on the halo appears as long strings of dots. In the nucleus prior to histone removal the genes are packaged and appear as a compact dot. When the histones are removed, the DNA is unpackaged (halos), resulting in a string of genes (dots). This technique allows the visualization of the relative positioning of genes to the nuclear matrix.

### FISH on extended DNA fibers

**Figure 5:**
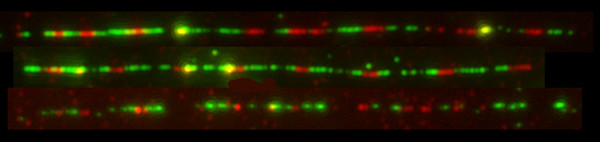
Extended DNA fibers isolated from wheat endosperm. Green, Alexa Fluor 488: genomic EcoRI fragments of HMW-1Dx5 (8.7 Kb) and HMW-1Ax1 (7.0 Kb) containing the promoter, coding and 3'flanking regions of the two high molecular weight glutenin genes transformed into *Triticum aestivum *L., cv Pro INTA Federal. Red, Cy3: pUC19 (2.7 Kb), vector backbone of the plasmids used for transformation.

Another development of the FISH technique is its application to extended fibers, which are usually prepared by detergent extraction of DNA from isolated nuclei. The naked DNA is then stretched by allowing it to run down a tilted slide. The sensitivity of FISH is greatly enhanced because without histones and other chromatin-bound proteins the DNA is more accessible to probes and detection reagents. Thus, with this method the detection of DNA targets as small as a few hundred base pairs becomes feasible [73]. The fiber FISH methodology has superior mapping resolution compared to interphase nuclei. For example, using probes hybridized to targets in the 45S rDNA genes of tomato it was possible to detect DNA target sequences as small as 700 bp [74]. The hybridization of T-DNA sequences in transgenic potato plants to extended DNA fibers revealed that T-DNA copies are closely integrated. Moreover, by using probes to T-DNA and vector sequences the composition and arrangement of inserts can be assessed [75]. FISH on DNA fibers has enabled (1) assessment of the effect of differences in probe length and the mapping of different probes relative to one another, providing detailed information on gene structure [76-82]; (2) analysis of the structure of repetitive DNA sequence families [74,81,83-85]; (3) analysis of transgenic DNA ([4,40,75]; Wegel, unpublished results]). We have used FISH to analyze the organization of a complex transgene locus comprising two different plasmids containing genomic fragments coding for two high molecular weight glutenins in *Triticum aestivum*. In figure 5, we show FISH with probes for the vector and the transgenic glutenin fragments on DNA fibers isolated from endosperm, visualized with a cooled CCD camera. The FISH signals show the transgene arrangement along the linearized chromosomal DNA demonstrating once again that loci generated by microprojectile bombardment are complex and contain numerous interspersions of genomic DNA.

## Simultaneous detection of transgenic DNA and RNA

The analysis of transgene expression is typically based on the steady-state level of mRNA or protein, which is extracted from the transgenic material. Methods to measure mRNA level include RT-PCR, Northern blotting, serial analysis of gene expression (SAGE) and microarray techniques. More recently, a technique based on Chromatin Immunoprecipitation named RNAPol-ChIP has been devised which allows analysis of real time gene transcription [86]. However, in all of these techniques the structure is destroyed and thus they are not suitable for examination of the expression of specific genes in small amounts of tissue, nor do they allow localization of the expression of a particular gene in specific cells or tissues. Moreover, results from these methods for gene expression reflect an average of expression from many cells. Therefore, techniques have been developed for microscopic visualization of RNA abundance and distribution, in particular the quantification and visualization of mRNA transcripts in individual cells [87-92].

Earlier observations with simultaneous DNA (DNA FISH) and RNA hybridization (RNA FISH) showed that in 90% of cells the gene was directly associated with an RNA track or focus. This observation provided confirmation that the transcript foci and tracks represented the sites of transcription, with the DNA positioned at or near one end of the RNA track [93,94]. Later, Van de Corput and Grosveld [95] were able to detect by RNA-FISH the primary transcripts of the human embryonic, fetal and adult globins in erythroid cells, and related expression patterns with other parameters such as cell type, cell cycle, replication, and stage of differentiation. More recently, the visualization of RNA has provided correlations between chromatin structure and gene expression upon transcriptional activation both in animal [92,96-99] and plant cells [15,16].

The ability to visualize the expression of many genes simultaneously within individual cells with high spatial and temporal resolution can help the understanding of relationships among genes in single nuclei. For example, Levsky et al. [89] showed that genes are not continuously transcribed, implying that individual cells have unique patterns of gene transcription. A similar observation was also reported by Osborne et al. [96] who showed that upon transcription distant genes co-localize to the same transcription factory whereas identical, temporarily non-transcribed alleles do not. These authors have used a combination of 3D FISH, immunofluorescence and chromosome conformation capture (3C) to assess the spatial organization for several genes in a mouse chromosome. The 3C technique allows determination of the relative frequencies with which different sites interact with each other [100]. Osborne et al. [96] determined the percentage of colocalization of the RNA-FISH and the corresponding DNA signals, as well as the colocalization of widely separated genes when these genes are being transcribed, and concluded that colocalization of genes is transcription-dependent. These studies also indicated that the most active genes undergo transcription on-off cycles, which correlate with occupancy of transcription factories during the on stage. Recently, by using a modification of 3C and FISH, Ling et al. [98] found the colocalization of distinct DNA segments located on different chromosomes. All these observations provide evidence to support the idea that genes are dynamically recruited to transcription sites, in agreement with the transcription factory hypothesis of Cook et al. (e.g. [101,102]). These mechanisms are likely to occur in the same way in plants although this has yet to be demonstrated. In another approach, Janiki et al. [92]used an inducible system in which a 200 copy transgene array of inducible transcription units was stably integrated into a euchromatic region of chromosome 1 in human cells. With this system they were able to observe that prior to transcriptional activation the transgene array is highly condensed and heterochromatinized. After the induction of transcription the RNA levels at the transcription site increased immediately. In plants, Wegel et al. [16] have used two wheat transgenic lines containing about 20 and 50 copies each of the HMW glutenin genes (HMW) which are developmentally activated in the endosperm at about 8 days after anthesis. They observed that, in non-expressing tissue, each transgene locus consists of one or two highly condensed sites, which decondense into many foci upon activation of transcription in endosperm nuclei.

The sensitivity of mRNA FISH is not very well defined. Van de Corput and Grosveld [95]estimated detection sensitivity as being of the order of 10 copies of a primary globin RNA transcript using oligonucleotide probes. They also demonstrated that different probes show different sensitivities even when the base composition is similar. This is probably due to the secondary structure of the RNA or its association with protein complexes which could render part of the RNA less accessible for hybridization.

In plants, the visualization of transcripts in different tissues has been accomplished by *in situ *hybridization of labeled single-stranded, antisense probes to specific mRNA sequences in semi-thin sections of plant tissue. This technique is especially valuable when a developmentally regulated and/or tissue-specific promoter is used to regulate transgene expression. For transgenic plants, a technique of two-color *in situ *hybridization using two gene-specific RNA probes labeled with different tags provides an extremely powerful tool for comparing the spatial expression patterns of two genes in a specific tissue/organ; for example, expression of the selective marker gene and the gene of interest.

**Figure 6:**
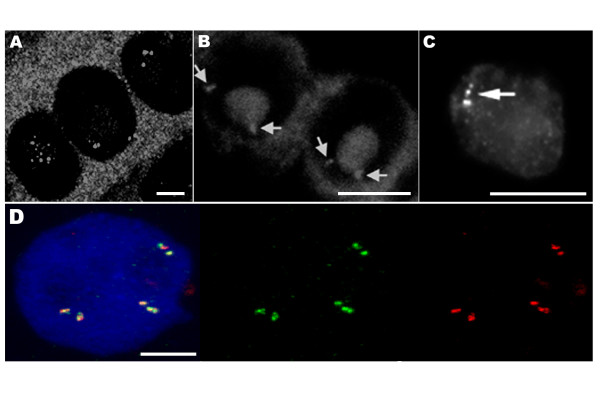
Transgenic RNA visualized in 3D interphase nuclei of root tissue sections from wheat (A), tobacco (B) and of an isolated tobacco nucleus (C). The wheat transgenic line illustrated in (A) carries five transgene copies at two sites on metaphase chromosomes as described in [2]. The tobacco transgenic line shown in B and C contains seven transgene copies. (D) Simultaneous localization of transgene loci and their transcript in a wheat endosperm nucleus 9 days after pollination. Nuclei counterstained with DAPI (blue) were hybridized with probes for the gene flanking regions and vector sequences of HMW-1Ax1 and HMW-1Dx5 to detect the locus (green, Alexa Fluor 488) and with an antisense probe for the 1Ax1 coding region to detect the transcripts (red, Alexa Fluor 633). The coding sequences of the two high molecular weight glutenin genes are highly homologous and cross-hybridize. The image is a projection of serial confocal sections. Section spacing, 0.6 μm. Bars, 10 μm.

In figure 6 we show localization of RNA in wheat root tissue sections (Fig. 6A), wheat endosperm (Fig. 6D), tobacco tissue sections (Fig. 6B) and tobacco nuclei (Fig. 6C). The sections were made in a vibratome, which preserves the cell and tissue structure well. In figure 6A, GUS transcript is shown in root tissue sections of a transgenic wheat line which contains two loci of the GUS transgene [2,14]. Along the wheat root section the RNA is particularly abundant in the xylem vessel cells which are clearly distinguished by a substantial increase in the size of the cell nucleus in comparison with the surrounding tissues. Endoreduplication has been shown to occur in these cells [103] and there is good evidence that after many endoreduplication events, the replicated chromosomes tend to remain together [103]. The increase in ploidy in these cells may be correlated with the higher transcription level seen in them. We have also investigated the localization of transcripts in whole tissue sections of root tips (Fig. 6B) and in isolated nuclei (Fig. 6C) of transgenic tobacco lines containing the GUS reporter gene. The GUS gene transcript shown in figure 6B has no intron. Thus, most of the FISH signal corresponds to nascent transcript at the locus and only relatively small pools of transcript are detected around the locus. A similar observation was reported by Wegel et al. [16], who localized intronless nascent HMW-glutenin transcripts within the nucleus of transgenic wheat and observed that the main RNA signal in the nucleus was always restricted to the close vicinity of the locus. Figure 6D shows a wheat endosperm nucleus hybridized with probes to visualize simultaneously the glutenin genes and their transcripts. The transcript was localized in the vicinity of the gene. In other experiments, we have detected small tracks emanating from the gene locus such as in the tobacco nucleus shown in figure 6C, which has also been observed in animal studies [93]. The *in situ *visualization of transcripts offers new insights into transgene expression analysis since variable expression levels can be detected *in situ *at cellular level. Moreover the physiological state of cells and the cell type within tissues can be correlated with a specific pattern of gene expression.

## Conclusion

In this report we present a practical assembly of useful techniques to visualize transgene organization in the interphase nucleus, when most genes are being actively transcribed and potentially interacting with each other. We have gathered data that contribute to a better understanding of: (1) the mechanisms involved in the stable and predictable expression of transgenes; (2) how different copies of the gene are positionally related; (3) the interactions between transgene copies integrated in different loci; (4) whether all transgene copies are active and how this is related to their position in the nucleus. All this information has a clear impact on the unraveling of structure-function relationships in the nucleus. In addition, the knowledge of transgene organization in the three dimensional interphase nucleus may also be crucial to better understand the relation between gene location and its activity. In plants, foreign DNA is thought to integrate randomly into the genome, which has been considered a major problem for plant transformation. Thus it is advantageous to select the lines of interest at an early stage by performing a full characterization of the transgene integration sites.

Transgene organization can be used as a tool to approach fundamental questions of nuclear organization, chromatin dynamics, and gene expression. We have shown DNA-FISH on four distinct states of decondensation, from well preserved 3D nuclei within intact tissue sections to the least compact state of chromatin: extended DNA fibers. Intact nuclei, either isolated or in tissue sections, preserve the three dimensional structure and provide the relative positioning of transgene loci. On the other hand, nuclear halos and DNA fibers allow for a higher resolution and finer detail of the locus structure. Together with chromosomal mapping of transgene loci using FISH, the methods described here provide a complete characterization of transgenic loci, which is fundamental to complement molecular analyses using PCR, sequencing and southern blotting.

We have shown that FISH to localize transgenic DNA can also be combined with *in situ *analysis of RNA, and therefore both gene and transcript can be seen in the same preparation. This type of experiment has only occasionally been carried out in plants as yet, but we believe that it will be more common in the future. It has been widely debated whether there is a correlation between the location of a transgene and regulation of its expression, and whether transgene copies integrated at different loci are all active. Further clarification of these issues needs efficient methods for *in situ *detection of the transgene within its genomic environment together with 3-dimensional microscopy and image analysis.

## Competing interests

The author(s) declare that they have no competing interests.

## Authors' contributions

APS carried out experiments on rice (Fig. 1 and Fig. 2B) and wheat (Fig. 2A and Fig. 6A) and co-wrote the manuscript. EW carried out experiments with DNA fibers (Fig. 5) and double DNA-RNA FISH (Fig. 6D). GCA and WFT guided experiments with transgenic tobacco lines and participated in writing the manuscript. ES provided the rice transgenic lines and contributed to the manuscript. PS supervised the work on rice and wheat and contributed to the manuscript. RA performed the experiments on tobacco (Fig. 2C, Fig. 3, Fig. 4, Fig. 6B and 6C) and co-wrote and coordinated the writing of the manuscript. All authors read and approved the final manuscript.
